# Epilepsy treatment over time: The chess game analogy for choosing, adjusting, and adding antiseizure medications

**DOI:** 10.1111/epi.70005

**Published:** 2025-11-07

**Authors:** André Palmini, André Gus, Lecio Figueira Pinto

**Affiliations:** ^1^ Department of Clinical Neurosciences, School of Medicine Pontificia Universidade Católica do Rio Grande do Sul Porto Alegre Brazil; ^2^ Porto Alegre Epilepsy Surgery Program Hospital São Lucas, Pontificia Universidade Católica do Rio Grande do Sul Porto Alegre Brazil; ^3^ Pediatric Epilepsy Surgery Program, Hospital Santo Antônio, Santa Casa de Misericórdia de Porto Alegre Porto Alegre Brazil; ^4^ Hospital de Clinicas, Universidade de São Paulo São Paulo Brazil

**Keywords:** antiseizure medications, chessboard game, epilepsy treatment, strategic decisions, treatment management over time

## Abstract

Recurrent seizures have such a negative impact upon the lives of persons with epilepsy that their prevention is a constant preoccupation of neurologists. Despite the large number of antiseizure medications (ASMs) currently available and of publications on their putative efficacy, adverse effects, and order of preference to start treatment, there is very little information on how to manage treatment over time. Here, we propose the analogy of a chess game to encourage neurologists to develop strategic decisions at each point in time to correctly manage ASMs toward the best possible degree of seizure control. We leverage that the strategy of the chess player must be adapted to each move of the opponent, to emphasize the need to individualize the treatment of epileptic seizures according to variations in response to ASMs that occur over time. The chess player calculates the best move at each point in the chess game just as the neurologist draws upon their knowledge and experience to select the best ASM regimen for a given patient and adjust it based on their "gestalt" of the patient's response to each treatment change. This gestalt, however, is firmly grounded in rational, flexible adaptations to ASM treatment protocols. Strategic decision‐making in several epilepsy scenarios is given to illustrate the utility of the chess analogy.

**Key points**

- There is a gap between the relatively abundant information on initiation or punctual add‐on modifications of treatment and the little guidance available on the complex process of following people with epilepsy over time, adjusting antiseizure medications as the situation evolves.
- It is crucial that neurologists develop strategies to deal with the many variables encountered as the treatment of epilepsy unfolds over time. Such strategies should derive from a combination of knowledge and experience, leading to the development of a "gestalt" of what may work best in each situation at each point in time.
- Treating epilepsy with ASMs is thus akin to playing chess—a game in which strategic decisions must be made at each point in time and constantly reevaluated to beat the opponent (in this case, the recurrent epileptic seizures).
- Neurologists young and old currently have a larger number of "pieces" at their disposal to move on the "chessboard" and a body of literature that allows a continuous build‐up of experience on how to manage the "game," that is, to increase the chances to develop successful treatment strategies over time, to eventually control seizures.

*It is not what the doctor knows, but what he does with what he knows that makes the difference* Hippocrates

## 1 CHOOSING ANTISEIZURE MEDICATIONS IS ONLY THE STARTING POINT: SEIZURE CONTROL OFTEN NEEDS TREATMENT ADJUSTMENTS OVER TIME

There is no shortage of publications on antiseizure medication (ASM) selection, adverse effects, probability of seizure control according to etiology, and comorbidities that might influence treatment of persons with epilepsy (PWEs).^1^, ^7^ However, the main problem is not how to start treatment, but rather how to make decisions over time when seizures recur, adverse effects arise, or circumstances in the life of the patient change. Because many difficulties arise in the strategic decisions to be made at every contact with the patient, we have found it useful to think of epilepsy treatment as a kind of chess game, in which each move must be carefully considered in building toward a successful endgame.^8^ The chess analogy may lead to approaches that combine well‐tested strategies taken both from the literature and the experience of the neurologist, while also encouraging flexible adaptation based upon mechanisms of action of the ASMs.^9^

Prevalence figures for epilepsy in the range of .5–1%^10^ neglect the fact that recurrent seizures in PWEs often hijack the minds and practical aspects of life of their "inner circle." For every PWE, there may be 2–10 people whose life is negatively impacted by the possibility that the patient may have a seizure at any moment.^11^ As such, controlling epileptic seizures is important not only to the patient but to a small community. However, studies on the efficacy and tolerability of ASMs, compared to placebo or to each other in monotherapy or add‐on trials, provide only general guidance on management^12^, ^14^ and do not address the best treatment choices for individual patients *at each point in time*. Moreover, these studies report an average of 50% seizure reduction compared to baseline with both old and new ASMs,^15^ and this may have limited impact upon quality of life.^16^

## 2 THE CURRENT LARGE NUMBER OF ASMs MAY BE A DOUBLE‐EDGED SWORD RESULTING IN THE FREQUENT USE OF POLYTHERAPY AND NEED FOR TREATMENT ADJUSTMENTS

Following PWEs for years is more complex than selecting a medication at the onset of treatment or establishing a given combination of ASMs at a single point in time (e.g., when seizures recur). Immediate and sustained seizure control is only one of several possible outcomes with a given ASM and treatment plan.^17^ The effect of the underlying etiology and seizure types on the probability of seizure control,^1^, ^18^, ^19^ patient sensitivity to adverse effects, drug–drug interactions, and comorbidities often lead to treatment adjustments.^2^, ^7^ These adjustments of treatment over time form the basis of the proposed analogy of ASM treatment to a chess game.

**FIGURE 1:**
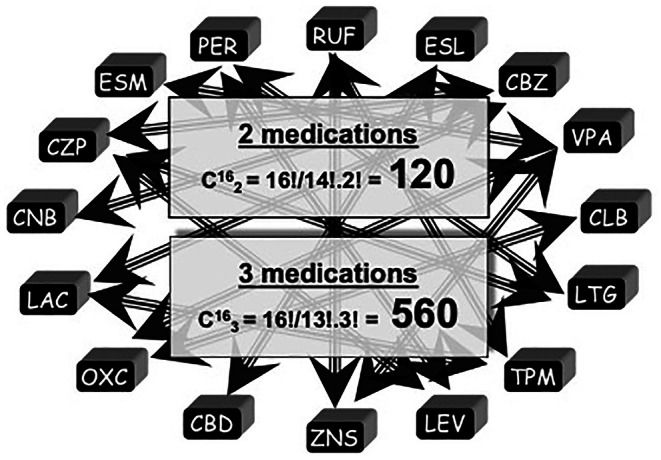
The pieces in the chessboard: 16 antiseizure medications and a combinatorial analysis of the large number of possible combinations in polytherapy. For the sake of the analogy, we kept the number at 16, yet the reader should consider that phenobarbital, phenytoin, and brivaracetam (not depicted) are also a frequently used antiseizure medications. CBD, cannabidiol; CBZ, carbamazepine; CLB, clobazam; CNB, cenobamate; CZP, clonazepam; ESL, eslicarbazepine; ESM, ethosuximide; LAC, lacosamide; LEV, levetiracetam; LTG, lamotrigine; OXC, oxcarbazepine; PER, perampanel; RUF, rufinamide; TPM, topiramate; VPA, valproic acid; ZNS, zonisamide.

The availability of an ever‐increasing number of ASMs has, in some respects, been a double‐edged sword in that it can add confusion in treatment choice and treatment adjustment. There are currently 30 ASMs available, making the choice of initial monotherapy somewhat complex, although guidelines and review papers provide some guidance.^4^, ^6^, ^20^ A recent study showed that only 36% of 443 patients with new onset focal seizures remained on monotherapy with their initial ASM after a relatively short period (mean = 2.1 years).^21^ In a much larger series of almost 1800 patients with new onset epilepsy, only 55% were seizure‐free on monotherapy for at least 1 year.^22^ Because seizures may recur after this time window, it is fair to say that more patients with epilepsy need polytherapy than remain on monotherapy in the long run. Applying combinatorial analysis to the 30 available ASMs, there are 435 possible combinations using two and 4060 using three ASMs. Even considering only 16 (the number of pieces on a side on a chessboard) ASMs, the number of possible combinations in duotherapy is 120 and combining three medications it is 560 (Figure 1).

When adjustments are needed in what we call the intermediate steps of the treatment, neurologists are left to their own devices, because there are no examinations or laboratory panels to assist in selecting the best next steps. It would be quite difficult to include the many variables affecting treatment outcome in epilepsy in guidelines.^19^ Treatment must be tailored for each PWE over time, as the challenges posed by seizure recurrence, adverse effects, comorbidities, and patient circumstances result in a complex equation that needs to be addressed at every consultation. When two or three medications are needed, the equation is much more complex, because these variables must be considered for each drug.

These issues are also relevant when neurologists entertain the possibility of epilepsy surgery. The probability of refractoriness when seizures persist after two trials of ASM treatment encourages referral to specialized centers for surgical evaluation.^19^, ^23^ Such referral and eventual epilepsy surgery does not obviate the need for careful ASM management, as a significant number of patients continue to need ASMs after operation.^24^, ^25^

## 3 THE CHESS GAME ANALOGY

Despite large interindividual variability in response to treatment and the many variables involved, we posit that managing ASMs is far from "shooting in the dark." Because most difficulties arise in the intermediate steps that demand strategic decisions at every contact with the patient, we propose an analogy to the game of chess (Table 1).

**TABLE 1:** Key features of the analogy between the treatment of epilepsy and playing chess.

On the chessboard	Playing the game	Analogy applied to epilepsy treatment
Pieces	Variable potencies to defend from and attack the opponent	ASMs
Movements of the pieces	Predetermined movement range and directions that make pieces useful in some contexts but not in others	Mechanisms of action of ASMs and putative efficacy in specific seizure types/epilepsies
Strategy of the player	Coordinated action involving multiple pieces	Medication schemes: ASMs, doses, titration
Loss of important pieces	Need to rethink the strategy due to loss of pieces	Adverse effects that suggest a given ASM should be stopped
Ineffective strategy	A chosen strategy may not work, allow advances of the opponent—and the player needs to change the strategy	Seizure recurrence despite ASM scheme; need to change the scheme
Opponent	The opponent may be more or less difficult to defeat	Epilepsy syndrome/etiology

Abbreviation: ASM, antiseizure medication.

The starting point is straightforward; ASMs are akin to the pieces on the chessboard. Each piece has specific movements—just as ASMs have specific mechanisms of action—and there are innumerable potential combinations of movements, like the many possibilities involved with managing ASM schemes: switch drug, increase dosage, reduce dosage, add another ASM, et cetera. In chess, movement of the pieces at each point is linked to the difficulties posed by the opponent,^8^ just as the response to treatment at each point demands adjustments in dosages and ASM schemes.^1^, ^19^ The strategy of the players (neurologists) is to decide which pieces to move and how to move, considering both the anticipated difficulties posed by of the opponent (i.e., how difficult seizure control is anticipated) and the development of the game (response to previous treatment schemes). The better players know their opponents—ie, the better neurologists know the severity of the epilepsy, its etiology, and other variables—the better their position to select the next moves (treatment adjustments). Seizure recurrences are akin to bold movements of the opponent, and as the game develops players may lose pieces, which are taken off the board, representing inefficacy or intolerable adverse reactions to ASMs—both situations demanding modifications of the strategy.

The pieces of the chess game are like weapons with distinct potencies both to defend and to attack. In our analogy, they refer to the comparative "potency" of an ASM (surrogates for expected or potential efficacy) toward a given seizure type in each epileptic disorder.^26^, ^27^ Comparative potency of ASMs is a difficult issue, because head‐to‐head studies often do not address the complexity of the PWE in front of us.^28^ In chess, some pieces, like pawns, are mostly supportive (yet, at times, crucial for strategy); others, like rooks, have a limited range of movement; whereas others, like the queen, are key to attack and defend. Similarly, ASMs have putative hierarchic positions in treatment selection, and neurologists should consider this in their treatment plans.^1^, ^7^, ^29^ Thus, the "gestalt" of the best move at each point in time is a mix of potency, tolerability, and experience with ASM schemes in specific epilepsy scenarios. Neurologists must develop this gestalt as they gain experience.

Chess often involves coordinated movements of more than one piece, and it is important to consider a variety of strategic options before each move (in epilepsy: increase dosage, switch to another high potency ASM, add a lower potency or supportive ASM, etc.). The skilled chess player focuses on the part of the board where most action is taking place, but at the same time is alert to all possibilities, including movements of pieces that are not at the center of the action (in epilepsy: interactions or side effects such as mood worsening in a patient with previous depression), to select the best move (strategy) at every step.

At times, the player loses important pieces and must change strategies to keep alive in the game. Following the emergence of severe or significant adverse events such as serious rash, weight gain, erectile dysfunction, intolerable somnolence, mood disorders, or cognitive difficulties, neurologists often must stop the responsible medication and reorganize the treatment strategy (Table 1).

One cannot overemphasize that knowing the rules of the game or how each piece moves is insufficient for chess mastery. Similarly, knowing treatment guidelines and ASM mechanisms is not sufficient for neurologists to master epilepsy treatment. Experience over time is crucial. We propose that this demands a delicate balance of "sticking" first to well‐tested and often effective and well‐tolerated ASM treatments for specific seizure types or epilepsy syndromes, similar to well‐practiced and often successful openings in a chess game. Then, as the game (the treatment response) evolves, the player (the neurologist) must make rational flexible adaptations to their strategy.

## 4 FIRST MOVE: WELL‐TESTED STRATEGIES: FROM CHESS TO ASM TREATMENT

Chess players study the game just as neurologists study epilepsy. Chess players test their game strategies just as we test our medication strategies. There are innumerable ways to play chess according to the movements of the opponent, and there are innumerable ways to initiate and stabilize ASM regimens considering the peculiarities of the opponent, in this case, the seizure types and underlying etiology in an individual PWE.

**FIGURE 2:**
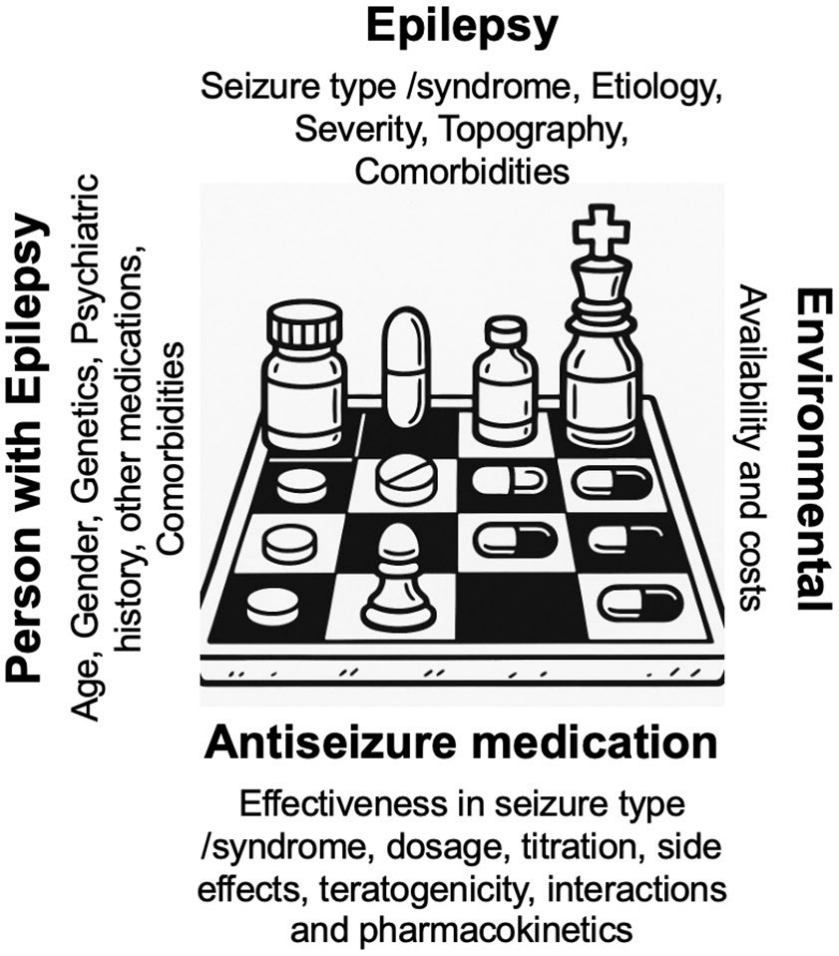
The antiseizure treatment (ASM) matrix. Note multilayered variables that must be considered in the selection of ASMs, related to the (1) epileptic disorder, (2) the patient, (3) the ASM, and (4) the environment.

The choice of preferred ASM schemes is made according to a matrix in which four groups of variables constantly intersect, related tothe epilepsy, the patient, and the medication (Figure 2). With experience, neurologists develop preferred ASM schemes (involving medications, combinations there of, dosages, titration schedules, side effect profiles, availability), according to variables related to the epilepsy (seizure type, etiology, severity, topography) and to the patient (age, gender, cognition, sensitivity to adverse effects, comorbidities, and other concomitant treatments). We suggest neurologists stick to well‐tested schemes first, just as chess players initiate the game with openings with which they feel most comfortable. The combination of seizure type(s) and etiology are the key factors to select not only the medication, but also ‐ crucially‐ the target dosage.^1^ This is of paramount importance, as it has been repeatedly shown that the chances of seizure freedom are greater with the first and second ASM regimen and decrease steeply with sequential medication changes, whether in mono‐ or polytherapy.^22^, ^30^ The three common situations below illustrate these issues:

1. In patients with focal seizures, a sodium channel blocker such as lamotrigine, lacosamide, or oxcarbazepine could be started, targeting intermediate dosages. Should seizures recur despite dosage adjustments, neurologists should use their experience (i.e., well‐tested schemes in their practice) and elect either to switch to another ASM in monotherapy, add an adjunctive medication such as clobazam, or else add a second high‐potency medication, usually with a synergistic mechanism of action (valproate, levetiracetam, or topiramate, for instance). Alternatively, some neurologists have found that adding a second sodium channel blocker can be effective.^31^ Finally, cenobamate is gaining traction as a very effective alternative for focal seizures and may soon climb the ladder of hierarchical choices.^29^ We propose that when there is a clear rationale, there are no right or wrong choices. However, the neurologist, like the chess player, should be ready to change the strategy according to the patient's response (seizure control, side effects), just as the chess player changes their strategy based on the opponent's move. A practical approach in many circumstances would be to select a medication "x" to start treatment, adjust the dosage, and add another ASM if that first drug was well tolerated but only partially effective. Should adverse effects occur with medication "x," then switch to monotherapy with drug "y" and follow the same path. Just as in chess there are certain "classical" moves at given moments in the game, neurologists can benefit from well‐tested strategies, such as adding clobazam to medications "x" or "y" above if the patient fails monotherapy. However, a well‐tested strategy in epilepsy treatment may not work for several reasons (efficacy, tolerability, etc.), just as a classical chess move does not always succeed, and treatment must be changed. That is where knowledge, experience, and gestalt intersect to guide the next treatment move (for instance, adding a medication with synergistic action or increasing the potency of a specific mechanism of action by using two ASMs with similar mechanisms).
2. For patients with epileptic encephalopathies associated with tonic and atonic seizures, many neurologists prefer the combination of valproate and lamotrigine, with or without the addition of a benzodiazepine. This combination is grounded in the literature, although other options are available.^32^ Some specialists prefer combinations including rufinamide, whereas others have had good experiences with topiramate or levetiracetam. More recently, particularly in children, cannabidiol is becoming a useful asset.^33^ All are suitable options in this scenario.^34^ We propose that no scheme is intrinsically better than the others, but it is crucial that neurologists use agents they know best, knowing that changes may need to be made downstream. These changes must be done with a clear rationale, just as the chess player reorganizes their strategy, or the game will be lost.
3. For patients with juvenile absence epilepsy, which despite its name often involves difficult‐to‐control generalized tonic–clonic seizures, there are several options. Based on a patient's gender and seizure evolution, valproate, lamotrigine, or levetiracetam monotherapy may be tested first, but despite adequate dosages a second ASM is often needed.^35^, ^36^ The choice of the latter is the quintessential example of prioritizing experience and well‐tested regimens, as it is imperative that the neurologist have a clear idea of ASM treatments that could deliver the best results. If patients mention that generalized convulsions are ushered in by a series of absence attacks, we have occasionally added ethosuximide, in the hope that controlling absences would reduce the progression to tonic–clonic attacks. In our hands, that strategy has not been very effective, akin to a chess player who at a specific point in the game decides to castle, only to see that it gave an unnecessary advantage to the opponent. After repeating a given strategy without success in consecutive matches, other moves will be chosen in midgame. Similarly, after several failures with other schemes, our experience guides us to prefer the combination of valproate and lamotrigine or valproate and levetiracetam, with careful dosage adjustments according to patient profile, particularly in the presence of psychiatric issues or in women of childbearing age. Other neurologists will have tested and adopted other preferred ASM combinations. There are no right or wrong choices. Ultimately, what works best in the hands of each neurologist is an acceptable choice, as long as the strategy is changed as needed.

Continuous literature review, exchanges with colleagues, and personal experience eventually dictate preferred strategies for each clinical epilepsy scenario. However, those may fail, and the ASM treatment must be changed, just as the chess player will need to adjust their strategy several moves deep following a bold move from the opponent.

## 5 NEXT MOVE: RATIONAL AND FLEXIBLE ADAPTATIONS

We are fortunate to have many ASM options at our disposal when treating a disorder as complex as epilepsy. However, having many treatment options does not mean they can be interchanged indiscriminately. Despite the number of available ASMs, seizure freedom has not increased over time,^22^ and the many options may at times be an easy trap, particularly in polytherapy schemes. Once a well‐tested strategy fails, there may be a temptation to select any other ASM and include that medication in the treatment plan, either as add‐on or replacement for another ASM. However, such choices should not be indiscriminate, and the principle of the chess game clearly applies. Every move should be thoughtfully considered, and the neurologist must have several well‐tested options as plans B, C, or D, to deal with treatment failures.

To summarize the recommended approach, neurologists should begin with their preferred treatment plans for common epilepsy presentations, update these with new knowledge as the field evolves, and test them sequentially, avoiding a checklist approach that selects a medication solely because it has not yet been tried in the patient. For example, cenobamate, a medication that may be more effective than others in some epilepsy presentations, is being used increasingly as a plan B.^29^ Plans B, C, and D for the most common epilepsy presentations may be unique to each neurologist and adapted to the circumstances of the patient at hand. An example of rational flexible adaptation is given in the following case.

### 5.1 An illustrative case: Treating epilepsy as playing chess

K.M.B. is a 26‐year‐old woman with autistic spectrum disorder who has intellectual deficiency and severe epilepsy. Seizures began at age 17 years and evolved with recurrent atonic, tonic, and tonic–clonic attacks. There is no presumed etiology. Gestation, delivery, and the neonatal period were uneventful, and full molecular genetic workup was normal. Electroencephalograms showed frequent bursts of generalized epileptic discharges and prolonged trains of polyspike–wave complexes interspersed with attenuation of the recording.

When first seen, she was having daily generalized tonic–clonic and atonic seizures, on a combination of oxcarbazepine 1800 mg/day, phenobarbital 200 mg/day, and clobazam 20 mg/day. Based on our experience,^32^ we initially instituted a regimen of valproate 1500 mg/day, lamotrigine 300 mg/day, and clobazam 20 mg/day. Generalized seizures went from daily to weekly, but because of repeated injuries she underwent selective posterior callosotomy,^37^ following which generalized tonic–clonic seizures were fully controlled and atonic attacks diminished in frequency from weekly to monthly. However, on two occasions she broke an ankle, and the negative impact of the few remaining drop attacks led us to change the ASM scheme. We first substituted clobazam for clonazepam and then added levetiracetam, both without significant change. We eventually slowly introduced cenobamate up to 300 mg/day. For the last 6 months, she has remained free from all seizure types with valproate 1500 mg/day, lamotrigine 300 mg/day, clonazepam 2 mg/day, and cenobamate 300 mg/day, without significant side effects.

This case illustrates a common problem in difficult‐to‐control epilepsies. Often, the ASM regimen chosen at the start of treatment may not control seizures, and adjustments must be made, following an evidence‐based rationale. In this case, when we first saw the patient, she was taking a combination of a sodium channel blocker; phenobarbital, a γ‐aminobutyric acidergic drug to which patients frequently develop tolerance; and a benzodiazepine. Because she had generalized seizures, we decided upon combined treatment with sodium valproate and lamotrigine. The former agent has proven efficacy for these seizures, and the latter is a sodium channel blocker with proven superior synergistic association with valproate.^38^ The clobazam was retained. As a second step, we simply changed from clobazam to clonazepam, as some of our patients with atonic seizures had better control with the latter. As a third treatment, we added levetiracetam, a broad spectrum ASM with a distinct mechanism of action, frequently used in generalized seizures.^27^ Finally, because some seizures continued despite these treatments and selective posterior callosotomy, we changed levetiracetam to cenobamate,^29^ carefully adjusted the dosages, and have succeeded in controlling the patient's disabling seizures.

In conclusion, to optimize the chances to checkmate a patient's epilepsy and offer better quality of life for PWEs and their inner circle, it is crucial that neurologists understand their opponent (i.e., the variables related to the epilepsy) in depth and integrate these variables with the "flow of the game." This flow refers to the treatment of epilepsy over time, for which there are no guidelines, and requires knowing which ASM or ASM combination usually works for each specific epilepsy presentation, taking into account the risk of adverse effects and comorbidities. The game requires the neurologist to know as well as possible the reach of each ASM and the ways to minimize unnecessary side effects, and to develop a "gestalt" of what could work best in each situation. Fortunately, there has never been a better time to exercise these abilities, and chess players would justly envy neurologists, as currently the latter have a larger number of "pieces" at their disposal, and much more literature on mechanisms of action of ASMs, potential successful "openings of the game" and, increasingly, on how to play with each piece as the game evolves. Therefore, chances to develop strategies and help patients are fortunately on the rise.

## AUTHOR CONTRIBUTIONS

André Palmini conceptualized the paper and wrote the first draft. André Gus and Lecio Figueira Pinto reviewed the manuscript and proposed substantial changes that led to the final form to be submitted.

## FUNDING INFORMATION

This study was supported by research productivity scholarship # 314492/2023–2–PQ 1A to A.P.

## CONFLICT OF INTEREST STATEMENT

A.P. has received honoraria for speaking and consultancy from Abbott, Adium, Aché, Apsen, Eurofarma, GreenCare, EaseLabs, FQM, Libbs, Novartis, Prati‐Donaduzzi, and UCB. A.G. has nothing to disclose. L.F.P. has received honoraria for speaking and consultancy from Abbott, Biolab, Eurofarma, Libbs, LivaNova, Prati‐Donaduzzi, Torrent, and UCB. We confirm that we have read the Journal's position on issues involved in ethical publication and affirm that this report is consistent with those guidelines.

## Data Availability

This is a position paper based upon data available in the literature. Data related to the patient whose case is illustrated are available on request from the corresponding author.
